# Right bundle branch activation during left bundle branch pacing: Marginal gains in left bundle branch pacing–optimized cardiac resynchronization therapy and the effects of atrioventricular delay dynamic optimization

**DOI:** 10.1016/j.hrcr.2023.12.005

**Published:** 2023-12-09

**Authors:** Julian Cheong Kiat Tay, Eric Tien Siang Lim, Terence Jiahui Wong, Jasper Jingyao Feng, Chi Keong Ching, Boon Yew Tan

**Affiliations:** ∗ Department of Cardiology, National Heart Centre Singapore, Singapore; † Cardiac Rhythm Management, Abbott Medical, Singapore; ‡ Prime Heart Centre, Gleneagles Hospital, Singapore

**Keywords:** Right bundle branch activation, Left bundle branch pacing (LBBP), Cardiac resynchronization, Noninvasive programmed electrical stimulation, SyncAV

**Key Teaching Points**

• Activation of the right ventricle (RV) during left bundle branch pacing (LBBP) is not well understood. Retrograde conduction from the left bundle branch to the right bundle branch (RBB) and septal myocardial capture and resultant indirect RV recruitment are potential mechanisms.
• We propose the use of noninvasive programmed electrical stimulation at varying outputs during unipolar and bipolar pacing configurations to elucidate the mechanism of RBB activation during LBBP.
• Understanding RBB activation patterns in LBBP would allow us to further optimize cardiac resynchronization therapy and potentially improve responder rates, not just in patients with LBBB but potentially in patients with RBB block or intraventricular conduction delay as well.

## Introduction

Conduction system pacing by positioning a deep septal lead that engages the left bundle branch (LBB) was first described by Huang and colleagues^1^ in 2017. LBB pacing (LBBP) as it is known today has since been shown to achieve physiological pacing with higher success rates and better pacing parameters compared to His bundle pacing.^2^ There is increasing evidence that LBBP is a viable alternative to traditional biventricular pacing (BVP) in patients with cardiac resynchronization therapy (CRT) indications. Compared to BVP, LBBP appears to be superior in its ability to narrow QRS duration (QRSd), improve ejection fraction (EF) and New York Heart Association (NYHA) class, reduce end-systolic volume, and increase number of super-responders.^3^ These benefits appear to be present even in patients with right bundle branch block (RBBB).^4^

The ability of LBBP to shorten QRSd in patients with RBBB has been previously reported.^5^ There are several potential mechanisms to account for this, and it centers on how pacing the LBB can also activate the right bundle branch (RBB):

(1) Although longitudinal dissociation of the LBB and RBB within the His bundle is well recognized, the presence of transverse interconnections between the LBB and RBB^6^ would allow LBBP to bypass a proximal block in the RBB. These transverse interconnections, however, would not be able to explain why native conduction fails to circumvent an RBBB. One possible explanation is the presence of bidirectional pathways within the atrioventricular (AV) node, one for antegrade and another for retrograde conduction.^7^ Conduction block may thus occur only selectively during native conduction antegrade to the RBB, but retrograde conduction during LBBP would circumvent the block.
(2) Myocardial capture of the septum at a sufficient output, either cathodally with the lead tip in the left ventricular (LV) septum or anodally with the lead ring at the right ventricular (RV) septum, overcomes the natural resistivity of fibrous sheaths in the longitudinal dissociated LBB and RBB in the His and allows for the RBB to be activated.

Assuming there is no conduction abnormality in the LBB or RBB, the surface electrocardiogram (ECG) manifestation of LBBP does not rely solely on how the LBB is engaged. During bipolar threshold testing, transition from nonselective LBB capture (ns-LBBP) with anodal capture to ns-LBBP without anodal capture, and eventually to selective LBB capture (s-LBBP), would correspond to an initial QS or Qr pattern in V_1_, to a broader QR/qR pattern when anodal capture is lost. This is followed by an even broader QRS and an rSR pattern in V_1_, and deeper S waves in leads I and V_6_ during s-LBBP. The V_6_ R-wave peak time (V6RWPT: time interval from unipolar pacing spike to R-wave peak in lead V_6_) remains unchanged as the LBB is captured throughout.^8^

The ECG manifestation of LBBP as described above is nonetheless an oversimplification. Both RV and LV activation is a fusion of conduction down the respective bundle branches and the septal myocardium activated via anodal or cathodal capture. The location of any conduction delay/block in the bundle branches would thus influence the overall morphology. For example, a block distal to the pacing site in the LBB would remain uncorrected during s-LBBP, but the left bundle branch block (LBBB) morphology is attenuated during ns-LBBP owing to LV activation from the cathodal capture of the LV septum.

The RV activation is more complex, as there is no direct RBB capture during LBBP. If there is retrograde conduction block proximal to the LBB pacing site, the RBB could still be activated via the slow transverse interconnections described above, or antegradely from a sinus beat through the AV node. The right ventricle could also be activated from anodal capture of the RV septum during bipolar LBBP at a sufficient output.

We can potentially elucidate the mechanism of RBB activation during LBBP using programmed electrical stimulation (PES). PES delivered deep-septally has been described as a method to confirm LBB capture.^9^ This maneuver should be delivered in a unipolar fashion to avoid anodal capture, as this may confuse any potential ECG changes during the study. We hypothesized that PES when delivered during ns-LBBP with anodal capture, ns-LBBP, and s-LBBP would provide valuable insights into how the RBB is activated.

We describe 2 patients who underwent LBBP, 1 with normal conduction and another with LBBB. PES was performed in patient 1 postimplant, and in patient 2 we demonstrated how understanding RBB activation during LBBP and SyncAV algorithm could correct LBBB.

## Case report

### Patient 1

A 77-year-old female patient presents with palpitations and recurrent syncope, which resulted in a traumatic subarachnoid hemorrhage. Telemetry demonstrated paroxysmal atrial fibrillation and recurrent postconversion pauses of up to 13 seconds. She underwent a pacemaker implantation to address her pauses.

A dual-chamber Abbott Endurity MRI^TM^ PM2172 (Abbott, Sylmar, CA) pulse generator (PG) was implanted. The leads comprised a 52 cm and a 58 cm Tendril^TM^ STS Model 2088TC (Abbott), respectively in the right atrium and deep septal position. The approach for deep septal implantation using the Abbott Agilis HisPro^TM^ Steerable Catheter has been described previously.^10^

**Figure 1:**
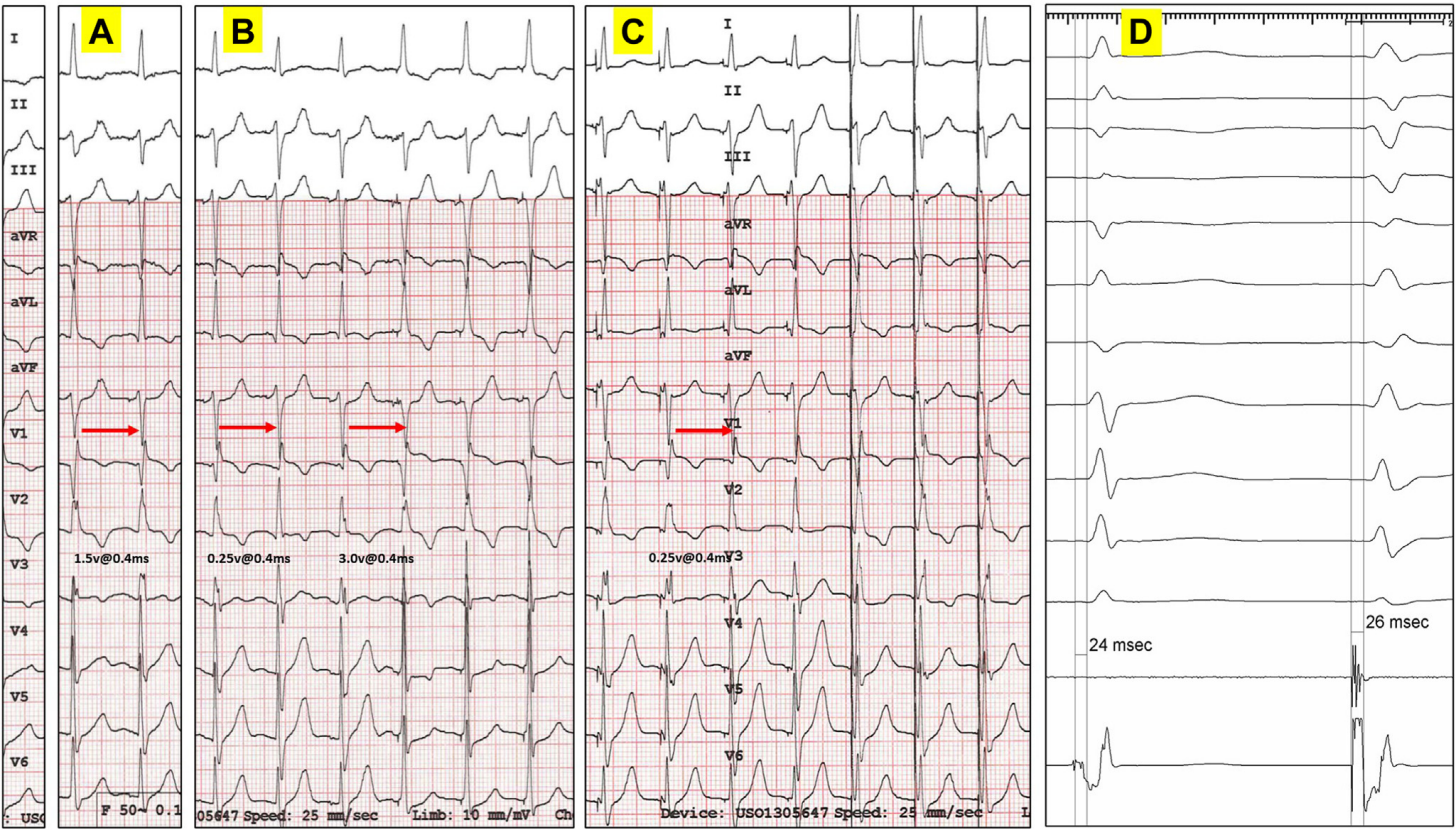
Bipolar and unipolar threshold testing post implantation. **A:** The first transition occurred at an output of 1.5 V @ 0.4 ms, corresponding with nonselective left bundle branch pacing (ns-LBBP) and anodal loss of capture (note a change in lead V_1_ from a Qr to a QR pattern). **B:** As output is further decreased, there is a second transition from a QR pattern to a qR pattern in lead V_1_, with an isoelectric interval, at an output of 0.25 V @ 0.4 ms corresponding with transition from ns-LBBP to selective (s)-LBBP, followed by the last 3 beats with a Qr pattern (safety pacing at nominal output 3.0 V @ 0.4 ms, ns-LBBP and anodal capture). **C:** Only 1 transition occurred at low output (anodal capture is not possible). In lead V_1_, transition from a QR pattern to a qR pattern with an isoelectric interval was noted at an output of 0.25 V @ 0.4 ms, corresponding with transition from ns-LBBP to s-LBBP, followed by the last 3 beats with a similar QR pattern (safety pacing at nominal output 3.0 V @ 0.4 ms, ns-LBBP without anodal capture). **D:** Left bundle branch potential (Po LBB) was demonstrated at implant. A similar interval was noted between Po LBB to QRS and stim-QRS with s-LBBP.

Postprocedure threshold testing was performed using bipolar and unipolar configuration. During bipolar threshold testing (Figure 1A and 1B), 3 distinct morphological changes were noted in lead V_1_, corresponding with ns-LBBP and anodal capture (Qr pattern in V_1_), ns-LBBP and anodal loss of capture (QR pattern, 1.5 V @ 0.4 ms), and s-LBBP (qR pattern with an isoelectric stim to QRS interval, 0.25 V @ 0.4 ms). Threshold testing was also performed using unipolar configuration (Figure 1C–1D). Two distinct morphologies were noted, corresponding with ns-LBBP (QR pattern) and s-LBBP (qR pattern with an isoelectric stim to QRS interval, 0.25 V @ 0.4 ms).

**Figure 2:**
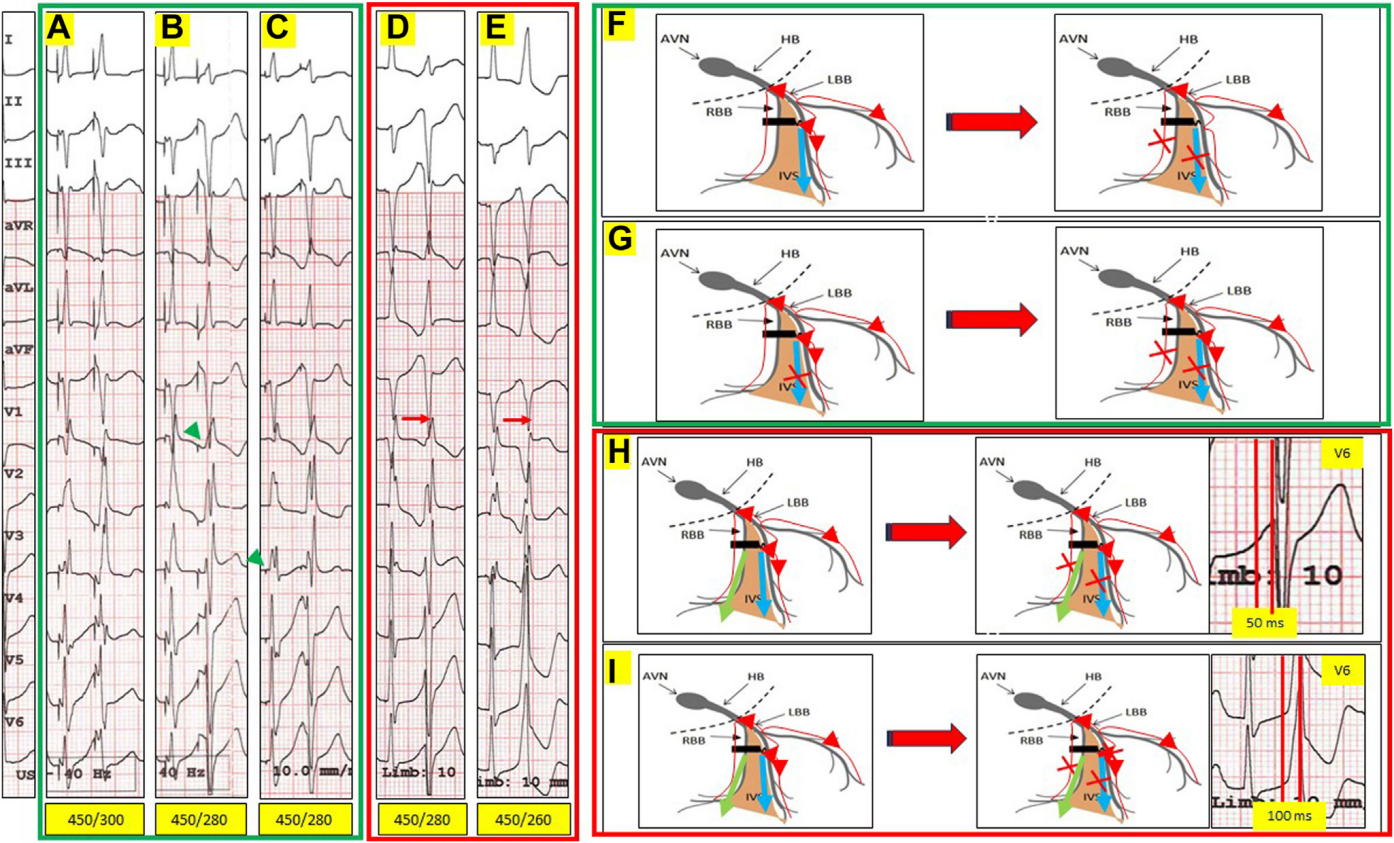
Unipolar and bipolar noninvasive programmed electrical stimulation (NIPS) using the implanted device. A drive train of 8 pulses was delivered at a cycle length (CL) of 450 ms (sinus CL was 550 ms), followed by S2 at 20 ms decrements. **A,B:** Unipolar NIPS output of 2.5 V @ 0.4 ms (nonselective left bundle branch pacing [ns-LBBP] without anodal capture). NIPS at 450/280 ms resulted in a change in morphology and an isoelectric line between the stimulation spike and QRS. The right bundle branch (RBB) and left ventricular (LV) septum in this instance have a similar effective refractory period (ERP). As a result, the extrastimuli S2 resulted in selective (s)-LBBP and RBB block (RBBB), with a clear isoelectric interval between stim to QRS (*green arrowhead*). (This is represented schematically in panel F). **C:** Unipolar NIPS output of 0.75 V @ 0.1 ms (consistent s-LBBP without anodal capture). An isoelectric interval was noted between the stimulus spike and the QRS with a qR pattern in V_1_ during the drive train. NIPS at 450/280 ms resulted in a change in morphology (similar RBBB morphology as in panel B; s-LBBP and RBBB). The qR pattern during s-LBBP is narrower (incomplete RBBB pattern), as there is retrograde conduction from the LBBP site and activation of the RBB via transverse interconnections. Conduction would be slower (hence a broader QRS) compared to normal conduction whereby both the RBB and the left bundle branch (LBB) are activated simultaneously from the atrioventricular node (AVN). (This is represented schematically in panel G). **D:** Bipolar NIPS at output of 2.5 V @ 0.4 ms (consistent ns-LBBP with anodal capture). NIPS at 450/280 ms resulted in a change in morphology (RBBB morphology; s-LBBP, RBBB, and anodal RV septal capture). In this instance, anodal RV septal capture ERP is greater than the RBB and LV septal cathodal ERP. The LBB continues to be captured, as evidenced by the short V_6_ R-wave peak time (V6RWPT) of 50 ms. (This is represented schematically in panel H). **E:** Bipolar NIPS at output of 2.5 V @ 0.4 ms (consistent ns-LBBP with anodal capture). NIPS at 450/260 ms resulted in another change in morphology in lead V_1_ (a LBBB morphology, Qr pattern; anodal RV septal capture alone, LBB ERP reached). The V6RWPT is now prolonged at 100 ms. (This is represented schematically in panel I). Blue arrows indicate cathodal LV septal capture; green arrows indicate anodal RV septal capture. HB = His bundle; IVS = interventricular septum.

To elucidate the mechanism of RBB and hence the RV activation during LBBP, unipolar noninvasive programmed electrical stimulation (NIPS) was first performed using the implanted device at an output of 2.5 V @ 0.4 ms (ns-LBBP without anodal capture) (Figure 2A, 2B, and 2F: green panel). NIPS was performed using a drive train of 8 pulses delivered at a cycle length of 450 ms (sinus cycle length was 550 ms), followed by S2 at 20 ms decrements.

As there is normal conduction physiology distal to the AV node in this patient, the unipolar paced QRS morphology at 2.5 V @ 0.4 ms (ns-LBBP without anodal capture) likely represents a fusion of (1) conduction down the LBB, (2) cathodal capture of the LV septum, and (3) retrograde conduction from LBBP site and activation of the RBB via transverse interconnections. NIPS at 450/280 ms resulted in a change in morphology and an isoelectric line between the stimulation spike and QRS (s-LBBP and RBBB, QRSd 130 ms). The effective refractory period (ERP) of the RBB and LV septum in this instance is similar and is greater than the ERP of the LBB.

Consistent s-LBBP can be achieved by unipolar pacing at an output of 0.75 V @ 0.1 ms (Figure 2C, 2G: green panel). The unipolar paced QRS morphology at this low output (QRSd 90 ms) likely represents a fusion of (1) conduction down the LBB and (2) retrograde conduction from LBBP site and activation of the RBB via transverse interconnections. This is evidenced by an isoelectric interval between the stimulus spike and the QRS, and a qR pattern in V_1_ during the NIPS drivetrain. As the ERP in RBB is greater than LBB, NIPS at 450/280 ms resulted in s-LBBP and RBBB (Figure 2B). This QRS morphology of qR in V_1_ and rS in V_6_ with QRSd 130 ms (s-LBBP with RBBB) is broader than s-LBBP without RBBB, as there is now only 1 activation wavefront down the LBB for both the RV and LV.

NIPS was also performed with bipolar pacing at an output of 2.5 V @ 0.4 ms (consistent ns-LBBP with anodal capture, Figure 2D, 2E, 2H, and 2I). The bipolar paced QRS morphology at 2.5 V @ 0.4 ms likely represents a fusion of (1) conduction down the LBB, (2) cathodal capture of the LV septum, (3) retrograde conduction from the LBBP site and activation of the RBB via transverse interconnections, and (4) anodal RV septal capture. From our earlier unipolar NIPS, we have demonstrated that the ERPs of the RBB and cathodal LV septum are similar and longer than the LBB ERP. Anodal capture of the RV septum is also likely to have a longer ERP than that of the cathodally captured LV septum.^11^

In Figure 2D and 2H, bipolar NIPS at 450/280 ms at an output of 2.5 V @ 0.4 ms resulted in a change in morphology in lead V_1_ (QR pattern to RBBB morphology with no obvious isoelectric line between stim to QRS). This likely represents s-LBBP (as evidenced by a short V6RWPT of 50 ms), RBBB, and anodal RV septal capture. In this instance, anodal RV septal capture ERP is shorter than the ERP of the RBB and the cathodally captured LV septum. Bipolar NIPS at 450/260 ms resulted in yet another change in morphology in lead V_1_ (Figure 2E and 2I; Qr pattern in V_1_, with prolonged V6RWPT of 100 ms). There is now a loss of LBB capture, and anodal RV septal capture alone remains.

### Patient 2

**Figure 3:**
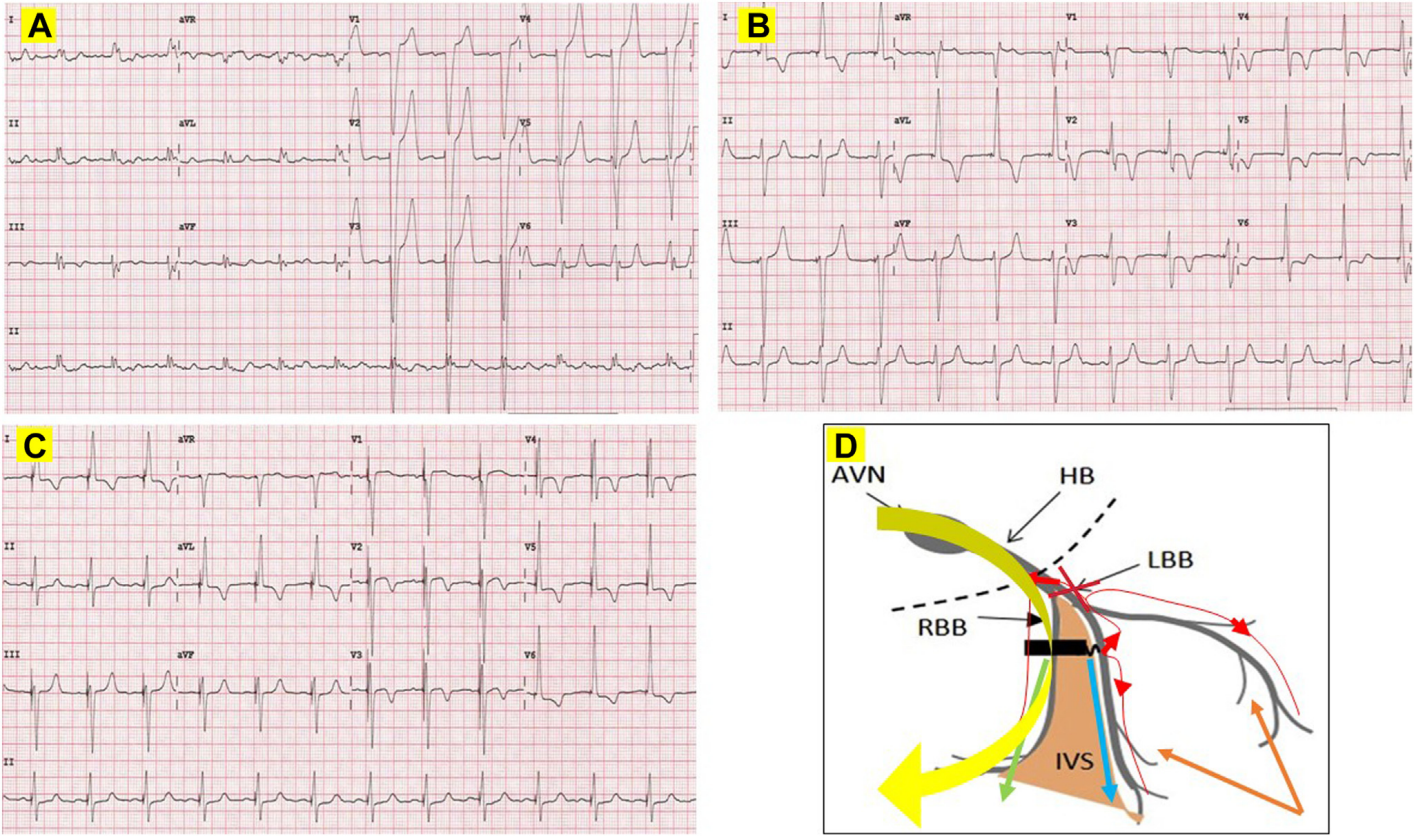
Baseline, postimplant left bundle branch pacing–optimized cardiac resynchronization therapy (LOT-CRT) and postimplant SyncAV LOT-CRT electrocardiogram (ECG). **A:** Baseline ECG for patient 2 showing normal sinus rhythm at a rate of 74 beats/min and left bundle branch (LBB) block. QRS duration of 170 ms, notching in leads I and aVL, QS pattern in leads V_1_ and aVR, and V_6_ R-wave peak time of 80 ms. **B:** Postimplant LOT-CRT ECG. Pacing in DDD mode with a short 80 ms atrioventricular (AV) delay. Left bundle branch pacing (LBBP) at an output of 2.5 V @ 0.4 ms (nonselective LBBP [ns-LBBP] and anodal capture) with an offset of +15 ms is followed by epicardial anterior lateral left ventricular (LV) pacing via the coronary sinus (CS) lead (M3-RV coil vector) demonstrated the narrowest fused QRS duration of 120 ms. **C:** Postimplant LOT-CRT final ECG. LBBP at an output of 2.5 V @ 0.4 ms (ns-LBBP and anodal capture) with an offset of +15 ms is followed by epicardial anterior-lateral LV pacing via the CS lead (vector: M3-RV coil). Sensed AV delay at baseline was measured at 250 ms. SyncAV delta of -80 ms resulted in further narrowing of the QRS to 100 ms. **D:** Schematic representation of likely activation wavefronts. The LV conduction abnormalities were corrected by a combination of (1) ns-LBBP (left bundle branch [LBB] capture and cathodal LV septal capture: QRS narrowed from 170 ms to 140 ms), and LV epicardial pacing via the CS lead (further shortening QRS from 140 ms to 120 ms). There is proximal LBB complete conduction block. SyncAV prolongs biventricular pacing AV duration dynamically, allowing for normal AV nodal conduction down the nondiseased right bundle branch (RBB), further shortening of the QRS to 10 ms. Blue arrow: cathodal LV septal capture; green arrow: anodal RV septal capture; yellow arrow: AV nodal conduction down the RBB with SyncAV; brown arrows: LV epicardial activation from the CS lead. AVN = atrioventricular node; HB = His bundle; IVS = interventricular septum.

A 70-year-old female patient with ischemic cardiomyopathy, EF 25%, and NYHA class III has recurrent heart failure admissions. Her baseline ECG shows sinus rhythm and LBBB (Figure 3A: QRSd 170 ms, notching in leads I and aVL, QS in V_1_ and aVR, and V6RWPT 80 ms). She was planned for CRT-defibrillator implantation.

A recent study has demonstrated that LBBP-optimized CRT (LOT-CRT) provides greater electrical resynchronization compared to conventional CRT.^12^ At 3 months, LOT-CRT confers better improvement in EF, LV end-diastolic diameter, N-terminal pro-B-type natriuretic peptide, and NYHA class compared to conventional CRT.^12^

LBBP was attempted first with a 58 cm Tendril STS Model 2088TC, as previously described.^10^ If LBBP narrowed the QRS sufficiently, a PG with a bipolar LV port at the header (vs a conventional quadripolar LV port) would be used, and the LBBP lead would be connected to this port. If LBBP fails to narrow the QRS sufficiently, then a conventional quadripolar CS lead would be advanced into a suitable CS branch, and LOT-CRT attempted. In this situation, 4 leads would be implanted, a right atrial lead, a DF1 RV defibrillation lead, a quadripolar CS lead, and the LBBP lead. The PG used in this situation would have a header that is compatible with a DF1 defibrillation lead. The pace/sense tip of the defibrillation RV lead would be capped, and the LBBP lead connected instead to the pace sense port for the DF1 RV defibrillation lead at the header.

**Supplemental Figure 1:**
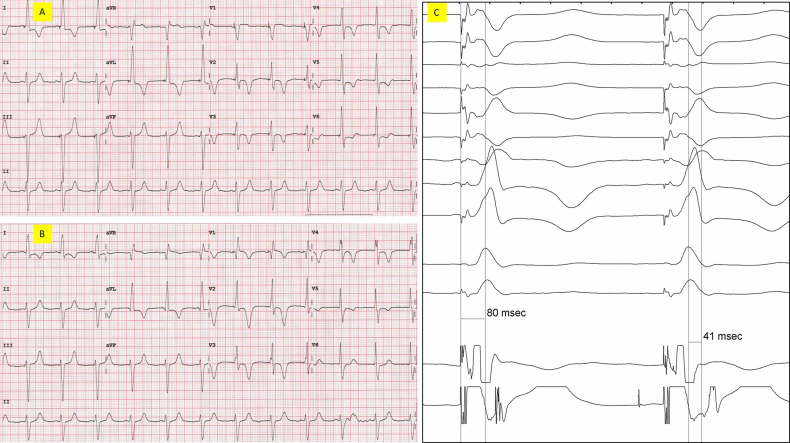
A) Post implant bipolar LBBP at an output of 2.5v@0.4ms demonstrating ns-LBBP with anodal capture. QS in V1, QRS duration 140ms, and V6RWPT 80ms. B) Post implant bipolar LBBP at an output of 1.0v@0.4ms demonstrating ns-LBBP without anodal capture. QR in V1, minimal change QRS duration, and V6RWPT remains at 80ms. C) Bipolar LBBP at an output of 2.5v@0.4ms demonstrating a V6RWPT of 80ms, and a V6-V1 interpeak of 40ms.

The LBBP lead was successfully positioned deep-septally (Supplemental Figure 1: V6RWPT 80 ms, V_6_–V_1_ interpeak 40 ms, QRSd 140 ms). The left posterior fascicle was targeted with resultant negative paced QRS in the inferior leads. During LBBP bipolar threshold testing, a change in V_1_ paced morphology was noted at an output of 1.75 V @ 0.4 ms, corresponding with loss of anodal capture (QS to qR pattern in V_1_). The QRS was shortened from a baseline of 170 ms to 140 ms with LBBP and anodal capture. We hypothesized that with the enlarged LV, there is likely further conduction delay distally that is not corrected with LBBP alone. A LOT-CRT was then attempted.

Using a short 10F sheath to gain access in the left axillary vein, a CPS Direct^TM^ Universal 135 sheath (DS2C020; Abbott, Sylmar, CA) with an inner self-selector CPS Aim^TM^ Universal CN-AL2 (DS2NO30-65; Abbott) was advanced into the CS main body using the over-the-wire technique. An anterolateral CS branch of suitable caliber was selected, and an Abbott Quartet^TM^ 1458Q/86cm (Abbott) quadripolar lead was advanced into this branch without difficulty. There were satisfactory pacing and sensing thresholds in all 4 poles of the quadripolar lead, with no phrenic nerve capture at high outputs. LV electrical delay (QLV) was also measured automatically using the device QLV algorithm, which measures from the first major deflection in the RV pace/sense terminal (in this case the LBBP lead) to the first major deflection recorded from each pole of the quadripolar lead. The M3 pole had the longest QLV at 107 ms, and a pacing vector M3-RV coil was chosen.

The best fusion and narrowest QRS was achieved by pacing the LBBP lead in a bipolar manner (ns-LBBP with anodal capture) 15 ms ahead of the CS lead using the M3-RV coil vector. The resultant ECG has a QS pattern in lead V_1_ and a QRSd of 120 ms (Figure 3B). This likely represents fusion of ns-LBBP and conventional CS epicardial pacing to correct LV activation delay. The RV activation, however, can only occur via anodal RV septal capture from bipolar LBBP. It is conceivable that complete conduction block in the LBB proximally has prevented retrograde conduction during LBBP, and hence is unable to activate the RBB.

An Abbott Tendril STS 2088TC/52cm (Abbott), followed by an Abbott Durata^TM^ 7122/65cm DF1 (Abbott) single-coil defibrillation lead, was implanted in the usual manner in the right atrium and right ventricle, respectively. All 4 leads were then connected into an Abbott Quadra Assura MP^TM^ CD3371-40C (Abbott) PG with a DF1 header.

This device is equipped with the SyncAV dynamic timing feature, which automatically adjusts the BVP pacing AV delay to create an optimal fusion between RV, LV, and intrinsic AV nodal conduction.^13^ Briefly, the device automatically extends the AV interval for 3 cycles after every 256 cycles to allow for the measurement of intrinsic AV conduction interval (measured AV conduction interval will be to the sensed activity in LBBP lead: LOT-CRT). A programmable SyncAV offset (default – 50 ms) is then subtracted from this measured AV interval and the result applied for the BVP paced AV interval of the following 255 beats, before the entire cycle repeats itself. The application of SyncAV in CRT has been associated with heart failure hospitalization reduction and a greater QRSd narrowing.^13^ Because there is likely bidirectional block proximal to the LBBP site, we hypothesized that SyncAV would still be able to shorten the QRS during LBBP, in that (1) antegrade block would prevent a stretched AV delay from interfering with LBBP capture, and (2) retrograde block in the LBB during LBBP would allow native antegrade conduction down to the RBB.

By stretching BVP pacing AV delay, SyncAV allows for intrinsic RBB conduction down a normally conducting RBB. In a patient with LBBB and a conventional CRT system, the RV activation would thus be a fusion of RBB conduction and RV apical pacing; and the LV activation, a fusion of RV apical pacing and CS epicardial pacing. We hypothesized that with SyncAV in LOT-CRT, further narrowing of the QRS can be achieved, especially if the site of block in the LBB is proximal to the LBBP site.^14^

Figure 3C and 3D demonstrates the final ECG in patient 2 with SyncAV on. The LBBP was paced at an output of 2.5 V @ 0.4 ms (ns-LBBP and anodal capture) with an offset of +15 ms (LBBP to CS lead: vector M3-RV coil). Sensed AV delay (atrial sense to LBBP lead) at baseline was measured at 250 ms. The SyncAV algorithm was switched on and various AV offsets tested to achieve the narrowest QRSd (programmable SyncAV offset range: -10 to 120 ms). The optimal offset of -80 ms was found to achieve the narrowest QRSd of 100 ms.

At 8 months, her NYHA class improved to I, and repeat echocardiogram showed EF normalization at 60% with improvement in LV end-diastolic diameter from 68 mm to 46 mm. All these features suggest that she is a “super-responder” to SyncAV LOT-CRT.

## Discussion

### Patient 1

We can appreciate distinct pacing morphologies correlating with ns-LBBP + anodal capture, ns-LBBP, and s-LBBP during unipolar and bipolar threshold testing. The RBB is activated by retrograde conduction during LBBP via transverse interconnections, accounting for the relatively narrow QRS during s-LBBP compared to a complete RBBB pattern if these connections did not exist. NIPS during ns-LBBP and s-LBBP in unipolar mode, and the resultant RBBB and isoelectric PR interval, allow us to conclude that the ERP for cathodal septal LV and the RBB are similar but greater than the LBB ERP. Bipolar pacing at a higher output introduces yet another wavefront, the anodal RV septal capture. By performing NIPs in bipolar mode, we demonstrate that ERP for the cathodally captured LV septum and RBB are similar but greater than the LBB ERP, which in turn is greater than the ERP of the anodally captured RV.

### Patient 2

Patient 2 has LBBB (QRS 170 ms). In a recent study,^15^ complete conduction block proximally in the LBB was found in up to 64% of patients with LBBB. While LBBP can potentially correct this, the resultant ECG will still have to contend with delayed RV activation, as the RBB is not directly paced, as well as any conduction delay in the distal LBB Purkinje system (QRS nonetheless shortened from 170 ms to 140 ms). The distal LBB Purkinje system conduction delay was mitigated by the introduction of an epicardially located CS lead (QRS shortened from 140 ms to 120 ms).

There is also bidirectional conduction block proximal to the LBBP site in patient 2, making it impossible for retrograde conduction to occur during LBBP. The site of bidirectional block in this instance is likely distal to any transverse interconnections to the RBB. The RBB remains intact and can conduct antegradely from the AV node. SyncAV prolongs the BVP AV duration sufficiently to allow this to occur, further shortening the QRS to 100 ms.

The mechanism of QRS narrowing during LBBP in patient 2 extends beyond just pacing the LBB. Optimal narrowing of the QRS during LBBP depends not only on our ability to overcome conduction abnormalities in the LBB (be it proximal or distal to the pacing site), but also on any potential RBB conduction abnormalities. Larger randomized trials would be necessary to draw definitive conclusions on the impact of such an approach, especially in patients with RBBB and intraventricular conduction delay.

## Conclusion

In LBBP, a standard 12-lead ECG and NIPS at varying outputs can help us understand how the LBBP is able to engage the conduction system beyond the LBB alone. These maneuvers can help us understand the level of block in the LBB and provide further evidence on the utility and effectiveness of dynamic BVP AV algorithms such as SyncAV during LBBP.

## Disclosures

The authors do not have any conflict of interest to declare.
